# Atherosclerosis as Extrahepatic Manifestation of Chronic Infection with Hepatitis C Virus

**DOI:** 10.1155/2016/7629318

**Published:** 2016-01-13

**Authors:** Theodoros Voulgaris, Vassilios A. Sevastianos

**Affiliations:** 4th Department of Internal Medicine, “Evangelismos” General Hospital, 45-47 Ipsilantou Street, 106 76 Athens, Greece

## Abstract

Chronic hepatitis C virus infection is associated with significant morbidity and mortality, as a result of progression towards advanced natural course stages including cirrhosis and hepatocellular carcinoma. On the other hand, the SVR following successful therapy is generally associated with resolution of liver disease in patients without cirrhosis. Patients with cirrhosis remain at risk of life-threatening complications despite the fact that hepatic fibrosis may regress and the risk of complications such as hepatic failure and portal hypertension is reduced. Furthermore, recent data suggest that the risk of HCC and all-cause mortality is significantly reduced, but not eliminated, in cirrhotic patients who clear HCV compared to untreated patients and nonsustained virological responders. Data derived from studies have demonstrated a strong link between HCV infection and the atherogenic process. Subsequently HCV seems to represent a strong, independent risk factor for coronary heart disease, carotid atherosclerosis, stroke, and, ultimately, CVD related mortality. The advent of new direct acting antiviral therapy has dramatically increased the sustained virological response rates of hepatitis C infection. In this scenario, the cardiovascular risk has emerged and represents a major concern after the eradication of the virus which may influence the life expectancy and the quality of patients' life.

## 1. Introduction

Over 160 million people worldwide are chronically infected with the HCV virus [1]. Besides the liver related complications of HCV infection such as liver cirrhosis and hepatocellular carcinoma, chronic infection is associated in several studies with extrahepatic disorders, including metabolic derangements. Though not many studies exist, robust data connect HCV infection with atherosclerosis and consequently its complications as stroke and coronary heart disease. In our days when the HCV infection can be treated in more than 90% of HCV infected patients, it is most important for clinicians to deal with the extrahepatic derangements which can diminish patients' life expectancy and alter their quality of life [2].

## 2. Pathophysiology Aspects

The pathophysiological basis of the evidenced correlation between HCV infection and atherosclerosis is incompletely understood. Chronic HCV infection is an inflammatory state not only affecting the liver. HCV infection represents a chronic inflammatory state where an imbalance between TH1 and TH2 is observed [3]. Studies have demonstrated that patients with chronic HCV infection exhibit higher IL-6 and TNF-alpha, INF*γ*, and IL-2 levels and a higher ratio of proinflammatory/anti-inflammatory cytokines [4]. Atherosclerosis is widely known to be a result of persistent inflammatory changes. HCV promoted inflammatory cytokines may contribute to the development of atherosclerosis through the enhancement of intracellular adhesion molecules, expression of anti-endothelium antibodies, and generation of oxidative stress (OXS) and insulin resistance (IR) [5]. What is more severe is that fibrosis and the associated cascade of proinflammatory and profibrogenic pathways generated in the liver might promote carotid atherosclerosis [6]. Furthermore, HCV RNA sequences have been isolated within carotid plaques and this in turn may suggest the possibility of an active infection of the carotid plaque itself [7].

Moreover, another important mechanism by which chronic HCV infection can promote the development of atherosclerotic plaques is its well described correlation with proatherogenic conditions such as insulin resistance [8, 9] and diabetes type 2 [10]. IR induces a broad range of toxic systemic effects, including dyslipidemia, hypertension, hyperglycemia, increased production of advanced glycosylation end products, increased inflammatory tone, and a prothrombotic and prooxidative state. Patients with IR are highly vulnerable to the development of accelerated atherosclerosis as well its clinical sequelae, including coronary artery disease and myocardial infarction, carotid artery disease, and ischemic stroke. Multiple explanations have been proposed in order to elucidate the mechanism of the development of IR in HCV infection. It seems more possible that both host and viral factors correlate to IR occurrence. Primarily it was assumed that chronic inflammation and the observed in HCV infection upregulation of inflammatory markers such as TNF-alpha and IL-6 and the deregulation of adipocytokines (leptin, adiponectin) were a leading step, but recent studies failed to prove this assumption. It is now believed that the HCV virus itself and moreover the HCV core protein are the main driving factor by their interactions with SOCS3 or SOCS7 expression and PPΑR-*γ* and PPAR-a [11, 12]. As far as type 2 diabetes and hyperglycemia is concerned its relationship with atherosclerosis is well established and its pathophysiological basis has been extensively studied. Oxidative stress, abnormal NO-mediated vasodilation, and increased macrophage lipid uptake, leading to foam cell formation, are only some features of the complex pathway by which hyperglycemia promotes atheromatosis [13].

**Figure 1:**
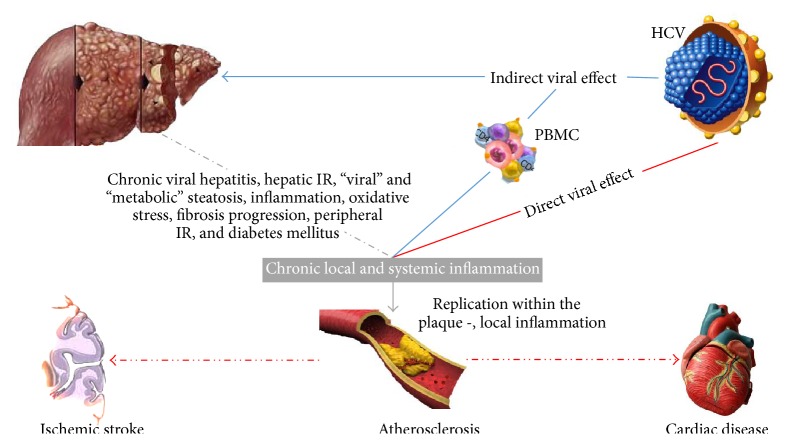
Possible mechanisms connecting HCV infection and cardiovascular disease. HCV is considered a “metabolic” virus and is associated with metabolic disorders, in particular insulin resistance and type 2 diabetes mellitus, which are proatherogenic conditions. By inducing hepatic injury and activating peripheral blood mononuclear cells (PBMC), HCV increases circulating levels of proinflammatory cytokines, leading to peripheral IR and hyperinsulinemia. Furthermore, a key feature of HCV infection is associated with hyperhomocysteinaemia, hypoadiponectinaemia, oxidative stress, lipid peroxidation, and all components of the metabolic syndrome. Therefore, “viral” induced and “metabolic” steatosis, together with the direct stimulus of increased insulin levels on hepatic stellate cells (HSCs) likely stimulate the progression of fibrosis within the liver parenchyma. Furthermore, systemic inflammation, the procoagulative state, and direct viral effects on the vascular wall may contribute to the development and progression of the atherogenic process.

Finally, liver steatosis, observed in HCV infection, and its association with hyperhomocysteinaemia are also factors predisposing to atherosclerosis [17]. Steatosis is a common finding in HCV infected patients especially among patients infected with Genotype 3 (GT 3) which seems to have a direct steatogenic effect as steatosis in infection with GT 3 is well correlated with the levels of intrahepatic viral replication [35]. Even if HCV related steatosis has not been proven to directly cause atheromatosis at least four studies have, independently of the metabolic syndrome, directly linked steatosis to atheromatosis [36]. It is not therefore irrational to hypothesize that this effect can be attributed to HCV related steatosis also. It should be mentioned that HCV patients tend to have a more favorable lipid profile possibly due to the straightforward interaction of the HCV virion, which uses the LDL receptor to infect hepatocytes, with the host lipid metabolism [35] (Figure 1).

## 3. HCV and Atheromatosis

In 2002 for the first time a study by Ishizaka et al. proposed a link between HCV infection and carotid atherosclerosis [14]. Since then several studies by various researchers, executed in different countries, provided evidence that HCV infection is independently associated with carotid plaques with a prevalence from 38% to 64% [37, 38] as also an independent predictor of increased carotid intimal medial thickness (IMT) (Table 1).

A study published in 2003 by Tomiyama et al., where 87 anti-HCV positives and 7427 anti-HCV negative subjects were enrolled, showed that HCV infected subjects had increased arterial stiffness compared to HCV negative controls [15].

Moreover a large Egyptian study by Mostafa et al. which included 329 anti-HCV positives and 725 anti-HCV negative patients showed that patients with active disease, when adjustment for known cardiovascular risk factor was executed, had a higher risk for atherosclerosis compared to subjects with past infection [16].

A recent study by Petta et al. not only confirmed the higher incidence of carotids plaques in HCV infected patients but also correlated the presence of carotid plaques with the severity of liver fibrosis as it was estimated by liver biopsy [6]. The study enrolled 174 GT 1 biopsy proven HCV patients and 174 control matched subjects. Multivariate logistic regression analysis showed that older age (odds ratio [OR] 1.047, 95% confidence interval [CI] 1.014–1.082, *P* = 0.005) and severe hepatic fibrosis (OR 2.177, 95% CI 1.043–4.542, *P* = 0.03) were independently linked to the presence of carotid plaques. In patients <55 years, 15/67 cases with F0–F2 fibrosis (22.3%) had carotid plaques, compared with 11/21 (52.3%) with F3-F4 fibrosis (*P* = 0.008). By contrast, in patients >55 years the prevalence of carotid plaques was similar in those with or without severe fibrosis (25/43, 58.1% versus 22/43, 51.1%; *P* = 0.51).

Finally, a study executed by Adinolfi et al. in Italy suggested that HCV-related steatosis is both a good marker for identifying atherosclerosis-prone individuals and an early mediator of atherosclerosis [39]. The writers came to the conclusion that HCV-related steatosis modulates atherogenic factors such as inflammation and the dysmetabolic milieu, therefore favoring the development of atherosclerosis. Once more, in this study, it was observed by the researchers that chronic HCV infection predisposes individuals to the premature development of atherosclerosis and advanced carotid changes.

On the contrary a small number of studies [19–21] failed to prove such an association, though it must be underlined that a meta-analysis executed by Huang et al., which included 11 studies among them the studies of Tien, Calsikan, and Masia, studies which failed to prove such an association, revealed that HCV infection is significantly associated with carotid atherosclerotic burden [40].

Taking into account the abovementioned data HCV infection must be considered as a risk factor for carotid atheromatosis. In the era of new and more efficacious treatments for chronic HCV infection the burden of the nonliver related complications of the HCV infection may become of great significance for the prior HCV infected patients' life expectancy. As a result of this, as it was also stated by Petta et al. [6], it may be indicated that HCV patients aged 55 or more, those with severe fibrosis, and those with HCV related liver steatosis should undergo ultrasonography screening for carotid atherosclerotic disease.

## 4. HCV and Stroke

Recent data have pointed out a correlation between HCV infection and increase risk for cerebrovascular disease. In a large study executed in the United States where 10,259 anti-HCV seropositive patients and 10,259 matched anti-HCV seronegatives were included, the Hazard Ratio (HR) of death from stroke was 2.20 [41]. In another study executed in Taiwan which enrolled 23,785 subjects (1,307 anti-HCV positive subjects) the HR for cerebrovascular death was 2.18 for seropositives to anti-HCV, compared to the seronegative patients of the study [42].

This positive correlation was also underlined in a recent study where 820 subjects were enrolled, where the multivariate analysis showed an OR of 2.04 for stroke among HCV patients compared to anti-HCV negative patients [26]. Finally, a large meta-analysis of the latest studies conducted in this field suggested that HCV infection [18] significantly increased the risk of stroke (OR = 1.97; 95% CI: 1.64–2.30).

Moreover it was proposed from a single study that HCV load is linearly correlated with the risk of stroke among HCV patients [43]. Of special note is a recent study conducted in Taiwan by Hsu et al. that came to the conclusion that not only did HCV infected patients have a 23% increased risk of stroke compared to age and sex-matched subjects without HCV infections but interferon-based therapy may reduce the long term risk of stroke in patients with HCV infection [44].

This data are furthermore supported by a recent study by Enger et al. [28] where it was demonstrated that HCV patients are in an increased risk of events such as unstable angina and transient ischemic attacks as it was pointed out in their study published in 2014 where 22733 HCV seropositives were enrolled.

To our knowledge only two studies, one including only 21 anti-HCV positive subjects, an obviously very small number of patients capable of extracting confident results [22], and another which was criticized because of the heterogeneity of the study population as far as age, sex, and hypertension status of the study subjects was concerned [45], failed to demonstrate such a positive correlation.

When all data are taken into account it can be safely argued that HCV infection increases the risk of cerebrovascular events and moreover data point to the direction that HCV eradication treatment can prevent these events, a fact highly important in our days when new and more effective treatment strategies against the HCV infection exist.

## 5. HCV and Cardiovascular Risk

Despite the existence of conflicting evidence, a link between HCV infection and increase cardiovascular risk can be discerned [5, 46]. The pioneer study in this field was published in 2004 by Vassalle et al. where the authors suggested that seropositivity represented an independent predictor for CAD with an odds ratio of 4.2 (95% CI: 1.4 to 13.0, *P* = 0.05) [23]. A large scale epidemiological study conducted in the United States by Butt et al. among (82083 HCV infected and 89582 HCV uninfected subjects) veterans over a 5-year period showed a significantly higher prevalence of cardiac disease among HCV infected patients [24].

In another study by Alyan et al. where 139 HCV seropositive and 225 HCV seronegative patients with angiographically documented CAD were enrolled, HCV infection was documented to be an independent predictor for increased coronary atherosclerosis, as demonstrated by higher Reardon severity score [25].

Recently a retrospective study including 78 HCV positive patients compared to 742 HCV negative subjects was executed, which observed higher ischemic heart events in the HCV positive patients than in the HCV negative patients (22% versus 13%, resp., *P* = 0.031) [26]. Additionally in a recent study where HCV monoinfected, genotype 1, naive, and nonobese (BMI < 30) patients and nondiabetics were included and compared to controls, an intermediate cardiovascular risk, as measured by the Framingham score, was observed [27].

As it was already stated Enger et al. [28] in a recent study came to the conclusion that HCV infected patients are in an increased risk of unstable angina. Moreover the results of the latest study published in 2013 by Satapathy et al. indicated that CAD is significantly more prevalent as also severe (stenosis > 75%) in HCV seropositive patients compared to age-, race-, and sex-matched controls undergoing evaluation by coronary angiogram for suspected CAD. The HCV infected patients were also presented, in a greater scale, with significant multivessel coronary artery disease (≥2 vessels). The authors notice that it is not clear whether the observed association between CAD and CHC infection is related to the known metabolic complications related to insulin resistance in patients with chronic HCV infection, or due to under treatment with antiplatelet and lipid-lowering agents because of concerns for gastrointestinal bleeding or hepatotoxicity [29].

Finally, a large recent study conducted in the US added more confirmatory data towards the direction of a positive association. Pothineni et al. in their study among a total of 8,251 HCV antibody positive, 1,434 HCV RNA positive, and 14,799 HCV negative patients came to the conclusion that there is an increased incidence of CHD events in patients with HCV seropositivity and the incidence is much higher in patients with detectable HCV RNA compared with patients with remote infection who are only antibody positive [30].

On the other hand, few studies [33, 34] and a recent review by Wong et al. [31] failed to demonstrate a clear-cut association between HCV infection and CAD. A large study executed in the UK by Forde et al. did not show any correlation between HCV and MI [32], though it must be underlined that there was a short period of follow-up of the subjects and moreover chronic HCV infection was poorly proved (the authors stated that they may have included patients who spontaneously cleared the HCV virus) and additionally it was a retrospective observational study where residual confounding by unmeasured confounders is possible. As far as the study of Arcari et al. which also failed to prove a correlation is concerned, not only was the sample size of the HCV infected patients too small but moreover there was no additional confirmatory PCR-RNA executed, a fact that may have further decreased the sample [33].

Even if some studies failed to prove this correlation it is not illogical to conclude that chronic HCV infection appears to be linked with excess cardiovascular risk (Table 2). Based on the abovementioned data HCV infection must be considered as a pre-atherogenetic state of an increased cardiovascular risk.

## 6. Conclusion

From a clinical point of view HCV infected patients not suitable for treatment or who have failed treatment options must be monitored for carotid atheromatosis, in order to prevent cardiovascular events. There are no formed guidelines but patients with severe liver fibrosis, HCV related steatosis, or of aged >55, who according to research data are of high risk for carotid atheromatosis, are the most eligible candidates for assessment of the existence of carotid atheromatosis.

Based on the bibliography, ultrasonography of the carotid arteries with IMT measurement should be offered to those HCV infected patients, in order to assess carotid atheromatosis as it is the test most commonly used in the studies conducted in this field.

In order to ameliorate HCV infected patients quality of life and to prevent extrahepatic complications, patients with well proven carotid atheromatosis should be offered primary prevention. It must be highlighted that not all patients are amenable of receiving primary prevention with antiplatelet therapy and lipid lowering agents. The risk of bleeding as also the liver related toxicity of lipid lowering agents must be balanced against the risk of a cardiovascular event.

Due to the lack of studies executed in this field, more data are needed in order to further specify which patients should be screened for carotid atheromatosis. The impact of HCV genotype as also that of the virus load should be further assessed.

In the era of new and more efficacious treatments for chronic HCV infection the burden of the nonliver related complications of the HCV infection may become of great significance for the prior HCV infected patient's life expectancy. As a consequence, it is a necessity to investigate if treatment does reverse the nonliver derangements such as carotid atheromatosis observed in HCV infected patients. Recent data have proven the reversion of liver fibrosis after the successful treatment of HCV infection [47, 48] and the diminished risk not only of HCC development but also of liver-related complications [49–51]. Moreover, the eradication of the virus inhibits the inflammatory cascade [52, 53]. It was already stated that patients with higher fibrosis scores showed a greater prevalence of carotid atheromatosis. It is not irrational then to hypothesize that carotid atheromatosis may also reverse the liver fibrosis and the superimposed inflammation tends to return to normal state but for the present time remains a scientific question whose answer must be provided by well-designed large randomized controlled studies.

## Figures and Tables

**Table 1:** Characteristics of studies associating HCV infection and atheromatosis.

Author, year, country	Study design	Association	Enrolled patients	Comments	Method of carotid atheromatosis assessment
Ishizaka et al., 2002 [14], Japan	Cross-sectional population based	Positive	4784/104 HCV infected	First study in this field, measuring IMT	Ultrasonography IMT measurement

Tomiyama et al., 2003 [15], Japan	Cohort study	Positive	7514/87 HCV infected	Increase arterial stiffness measured by pulse wave velocity	Pulse wave velocity

Mostafa et al., 2010 [16], Egypt	Cross-sectional	Positive	329 anti-HCV positive/724 anti-HCV negative	Patients with active disease had higher risk compared to past infection	Ultrasonography IMT measurement

Petta et al., 2012 [6], Italy	Case control	Positive	174 genotype 1 infected/174 controls	Association between fibrosis and the presence of plaques	Ultrasonography IMT > 1.3 mm

Adinolfi et al., 2012 [17], Italy	Case control	Positive	803/326 HCV infected	Association between HCV steatosis and atheromatosis	Ultrasonography IMT: >1 mm or plaques ≥ 1.5 mm

Huang et al., 2013 [18], China	Meta-analysis	Positive		Strongly correlates HCV infection to carotid atheromatosis	

Masia et al., 2011 [19], Spain	Cohort study	Negative	138 HIV/63 HCV/HIV coinfected	No matching between exposed and control patients for any variable	Ultrasonography IMT > 1.0 mm

Caliskan et al., 2009 [20], Turkey	Prospective 59 months follow-up	Negative	36 HCV infected/36 controls	No matching between exposed and control patients for any variable	Ultrasonography IMT > 1.0 mm

Tien et al., 2009 [21], USA	Cross-sectional	Negative	1675/53 HCV monoinfected	HIV/HCV coinfection may be associated with a greater risk of carotid plaques	Ultrasonography Focal CIMT > 1.5 mm in any of the imaged segment

Völzke et al., 2004 [22], Germany	Cross-sectional	Negative	4310/15 HCV infected	Very small number of HCV infected patients	Ultrasonography IMT measurement

**Table 2:** Characteristics of studies associating HCV infection and CAD.

Author, year, country		Association	Subjects	Comment
Vassalle et al., 2004 [23], Italy	Case control	Positive	491 with CAD (6.3% HCV seropositive)/195 controls (2% HCV seropositive)	First study that suggested HCV seropositivity as one of the risk factors affecting the onset and development of CAD

Butt et al., 2009 [24], USA	Prospective observational cohort study, 5 yr follow-up	Positive	82,083 HCV infected/89,582 HCV uninfected subjects	HCV infection is associated with a higher risk of CAD after adjustment for traditional risk factors

Alyan et al., 2008 [25], Turkey	Case control	Positive	139 HCV seropositive/225 HCV seronegative patients	HCV infection is an independent predictor for increased coronary atherosclerosis (higher Reardon severity score)

Adinolfi et al., 2013 [26], Italy	Retrospective cohort study	Positive	820/78 HCV infected	A secondary analysis showed that HCV patients had higher prevalence of past ischemic heart disease

Oliveira et al., 2013 [27], Brazil	Cross-sectional comparative study	Positive	62 HCV infected/11 controls	HCV infection was related to higher FRS as well as to higher pro-anti-inflammatory cytokine profile

Enger et al., 2014 [28], USA	Retrospective matched cohort study	Positive	22,733 HCV infected/68,198 comparators	Arterial events, especially unstable angina and transient ischemic attack, were more frequently seen in HCV patients

Satapathy et al., 2013 [29], USA	Retrospective, case control study	Positive	63 HCV infected patients/63 controls	The prevalence and severity of CAD were higher in HCV patients who were evaluated for CAD by angiogram compared with matched non-HCV patients

Pothineni et al., 2014 [30], USA	Retrospective cohort study	Positive	8,251 HCV antibody positive/1,434 HCV RNA positive/14,799 HCV negative patients	Increased incidence of CHD events in patients with HCV seropositivity; the incidence is much higher in patients with detectable HCV RNA compared with patients with remote infection who are only antibody positive

Wong et al., 2014 [31], USA	Systematic review	Unclear association		Systematic review

Forde et al., 2012 [32], UK	Retrospective, population cohort, 3.9 yr follow-up	Negative	4809 HCV seropositive/71 668 seronegative	No correlation between HCV and MI but short period of follow-up of the subjects and moreover chronic HCV infection was poorly proved

Arcari et al., 2006 [33], USA	Case control	Negative	292 case subjects/290 controls, overall 52 HCV positive	No association was found between HCV positivity and acute myocardial infarction

Momiyama et al., 2005 [34], Japan	Case control	Negative	524 with CAD (3.4% HCV infected)/106 controls	
